# Rethink nutritional management in chronic kidney disease care

**DOI:** 10.3389/fneph.2023.1108842

**Published:** 2023-01-27

**Authors:** Fangyue Chen, Krit Pongpirul

**Affiliations:** ^1^ Department of Preventive and Social Medicine, Faculty of Medicine, Chulalongkorn University, Bangkok, Thailand; ^2^ Thailand Public Health Research Fellowship, Health Education England, Leeds, United Kingdom; ^3^ School of Public Health, Faculty of Medicine, Imperial College London, London, United Kingdom; ^4^ Department of International Health, Johns Hopkins Bloomberg School of Public Health, Baltimore, MD, United States; ^5^ Clinical Research Center, Bumrungrad International Hospital, Bangkok, Thailand

**Keywords:** chronic kidney disease, digital health, wellness, clinical nutrition, lifestyle, artificial intelligence

## Introduction

“Today, your kidney function is 80% of your baseline. Drink more water.” “You have reached your daily allowance for salt intake.” “You should see a doctor about your kidneys in five years.” Mr. K adjusted his wristband while reading the notifications on his smartwatch.

As we move towards 2030, the world has committed to reducing premature mortality from noncommunicable diseases by one third as part of sustainable development goals (SDGs) (1). Since 1990, while cardiovascular disease has seen a decline in mortality rate by 30.4% and cancer by 14.9%, chronic kidney disease (CKD) has hardly seen a change (2, 3). Estimated to affect 9.1% of the global population, CKD is associated with 1.2 million deaths and 1.4 million additional deaths related to cardiovascular disease. This is higher than the number of deaths from tuberculosis (TB) or the human immunodeficiency virus (HIV) (2). Despite this reality, only 36% of countries have recognized CKD as a health priority (4).

Chronic kidney disease is an umbrella term that encompasses a highly heterogeneous group of conditions, including primary chronic diseases, diabetes, and hypertension, which can lead to abnormalities in kidney structure or function that persist for more than 3 months (5, 6). CKD is often an incidental finding among individuals who are primarily asymptomatic at an early or even later stage. Unpredictable in its progression rate, once the disease reaches its end stage, life-sustaining treatment, renal replacement therapy (RRT), can place a significant burden on the quality of life of the individual. Moreover, RRT is costly. Globally, 47-71% of patients who needed RRT could not receive it due to cost and lack of government support, causing them to die prematurely (7).

## Chronic kidney disease under the “illness” model

The World Health Organization (WHO) definition of health emphasizes health rather than the absence of disease (8). While public health policies have increasingly directed efforts toward disease prevention, CKD care remains primarily centered on disease management. Attempts have been made to improve disease prediction and surveillance, notably through the increasing number of risk prediction tools developed to identify risk factors for CKD and predict the development and progression of CKD. Key examples include a 5-year CKD risk prediction tool developed by Nelson et al., using data from 34 multinational cohorts as part of the CKD Prognosis Consortium (9). Toward the end of the disease progression, Tangri et al. developed the kidney failure risk equation (KFRE) derived from a predictive model for the progression of CKD to kidney failure (10). The tool has been validated in 31 multinational cohorts and incorporated into clinical guidelines as a critical decision-making aid (11). GFR and albuminuria levels have also been incorporated into risk prediction instruments for cardiovascular diseases (12, 13).

These prediction tools are helpful to inform an individual how likely it is that they will develop the disease or reach a critical stage at a chosen time point. However, candidate predictors are mainly collected during the snapshot of clinical contacts, such as demographic variables, comorbidities, and laboratory variables. Nelson et al. noted the potential contribution of other covariates predictive of CKD (9). What about our daily lifestyle behaviors? How do they play a role in contributing to disease development and progression? These questions remain largely unanswered. Understanding the critical daily modifiable factors will mean that people will not only know when they may need medical support but, more importantly, they can take ownership of monitoring and managing their state of health through what they do daily.

## The shift toward “wellness”

As critical organs responsible for excretion and homeostasis, the kidneys fine-tune bodily fluid compartments and compositions in response to what we consume through our dietary intake. CKD is associated with a disordered nutritional status. Current dietary recommendations for patients with CKD that aim to delay disease progression toward dialysis shed light on potential nutritional risk factors that lead to the development of CKD (14). Considering individual dietary constituents, experimental evidence has suggested a possible exacerbating effect of a high-protein diet, which can cause glomerular hyperfiltration and pro-inflammatory gene expression (15–17). On the other hand, a higher potassium excretion level is associated with a lower probability of renal complications in individuals at increased risk of cardiovascular disease or diabetes (17). In a systematic review, Kelly et al. found 57 modifiable dietary factors that showed potential associations with incident CKD. The evidence behind each dietary factor is scarce, so that only nine factors could be pooled for meta-analysis, and were found to be consistently associated with a lower risk of CKD. These include a higher vegetable intake, a higher potassium intake, and a lower sodium intake. Factors such as cereal fiber, coffee, and nitrate consumption can decrease the risk, as observed in one or two studies (18).

The accelerating development of data mining and machine learning techniques has unleashed the potential to discover complex nonlinear patterns within a dataset of increased volume, variety, and veracity, generating new knowledge and insight. Peng et al. explored lifestyle risk factors for CKD using association rule mining to analyze questionnaire data from 450,000 individuals (19). Interestingly, the recommended lifestyle modifications for CKD differed according to the comorbidities of the individuals. Those with cardiovascular disease should focus on increasing aerobic capacity. On the contrary, those with chronic obstructive pulmonary disease (COPD) or rheumatoid arthritis should emphasize high dietary fiber intake and moderate intensity exercise. This highlighted the importance of a personalized approach to lifestyle changes. The study uses the 2017 Behavioral Risk Factor Surveillance System (BRFSS), an annual health-related telephone survey. The study author acknowledged limitations that include a bias towards white race and male gender, which limits the generalizability of lifestyle interventions, as well as recall bias and a low response rate. Similarly, Luo et al. used machine learning modeling to establish a risk identification system for CKD by incorporating key risk factors that contribute to the development of CKD (20). The study recommended a healthy lifestyle consisting of whole grain bread, oat cereal, and muesli, along with walking and moderate physical activity. Biscuit cereal, processed meat, and tea > 4 cups/day increase the risk of CKD. Individuals with the best lifestyle score had a 70% lower chance of CKD than those with the lowest lifestyle score. Although the study is strengthened by the use of a large population of 470,778 from the UK Biobank and a long follow-up time (median of 11 years), most participants have adopted a Western lifestyle, calling for a greater need to study cohorts with greater ethnic diversity from Asia, Africa, and other areas, whose lifestyles differ, and can impact differently on CKD development and progression.

## Mobile apps, wearables, and artificial intelligence

The availability of relevant data is essential to explore the association between lifestyle-modifiable factors and CKD. Most of the data used in nephrology research are collected through clinical encounters, such as electronic health records and national insurance claim databases. They often contain minimal information on the individual’s daily lifestyle behaviors. Dedicated databases such as the Biobank cohort in the United Kingdom assess lifestyle behaviors through a questionnaire that relies on the individual’s reporting of lifestyle patterns, which is prone to recall bias and inaccuracies (20). How do we obtain information on the daily behaviors of the individual?

Mobile nutrition applications have been developed to help patients with CKD monitor and adhere to nutritional recommendations from healthcare professionals (21). For example, a diet intake monitoring app for adults receiving hemodialysis has a scanner feature that allows individuals to enter their nutritional intake, thus monitoring their daily dietary behaviors and making recommendations accordingly (22). Although non-compliance is the most common barrier to its use and continuation, these applications offer helpful solutions to collect data on individual daily lifestyle behaviors and support them in managing their dietary intake.

Wearable devices, sensor devices that can be attached to clothing or worn as an accessory, may circumvent non-compliance, unlock the potential to generate real-time information on health status, and empower individuals to manage their health (23). A recent systematic review of the influence of wearables on health care outcomes in chronic diseases found a mixture of positive and neutral results, depending on the targeted disease and the type of wearables used (23). One study on chronic kidney disease by Li et al. demonstrated positive outcomes after introducing a digital platform for patients with CKD that combined a wearable device that tracked steps, calories and sleep, a mobile health management platform that collated data followed by the wearable, and a dietary feature that allowed participants to upload photos of their daily meals (24). The randomized controlled trial has shown that after 90 days of intervention, individuals showed a significantly slower decrease in eGFR, along with higher quality of life scores, self-efficacy, and self-management scores. However, the study is limited by a small sample size of 49 individuals given the available number of wearable devices, and a short follow-up of 3 months only. In the study, researchers provided participants with personalized lifestyle recommendations after reviewing live data; artificial intelligence has shown promise in taking on this role in becoming the patient’s personalized advisor that can support their self-management (25).

The wealth of data that wearable devices record on the lifestyle behaviors of individuals can be used to generate new evidence. For example, Kim et al. identified key lifestyle factors associated with the severity of diabetes by applying the machine learning-based clustering technique on health-related behavior data recorded in real time through a Fitbit device (26). The study found that sleep habits, quality, and duration were the main determinants of a healthy lifestyle. Future-ready wearables can record a more excellent range of physiological and biochemical parameters, such as precise measurement of nutritional intake (27). Overall, mobile health allows real-time physiological monitoring, personalized digital diagnostics, and daily management.

## Discussion

Current technological advancement points toward a not-so-distant future, where individuals can monitor their health, including their kidneys, and manage their lifestyles according to recommendations based on the evidence generated from their individualized live data. The question is: how far are we willing to go?

It could be argued that we are medicalizing every moment of our daily behavior, yet if we shift from an illness-based to a wellness-based model, monitoring our kidney health will be akin to our current pedometer on our smartwatches and calorie counters at the gym. There must be a stringent governance structure and regulatory mechanisms to ensure the safety and quality of these potential futuristic tools and protect individuals against data misuse (28). Individualized self-management can transform the structure and function of current health systems. However, the current reality is that although CKD is an important public health issue with significant innovation potential, only a few countries have recognized it as a health priority (4). Greater national and international engagement and public, private, and academic collaboration will be required to improve and transform CKD care.

## Author contributions

FC, KP: Conceptualization. FC: Writing – Original Draft Preparation. FC, KP: Writing – Review & Editing. KP: Supervision and guarantor. All authors contributed to the article and approved the submitted version.
